# Impact of a national tsetse control programme to eliminate Gambian sleeping sickness in Uganda: a spatiotemporal modelling study

**DOI:** 10.1136/bmjgh-2024-015374

**Published:** 2024-10-30

**Authors:** Joshua Longbottom, Johan Esterhuizen, Andrew Hope, Michael J Lehane, TN Clement Mangwiro, Albert Mugenyi, Sophie Dunkley, Richard Selby, Inaki Tirados, Steve J Torr, Michelle C Stanton

**Affiliations:** 1 Department of Vector Biology, Liverpool School of Tropical Medicine, Liverpool, UK; 2 ARC-Onderstepoort Veterinary Research, Private bag x5, Pretoria, South Africa; 3 Bindura University of Science Education, Bindura, Zimbabwe; 4 Coordinating Office for Control of Trypanosomiasis in Uganda, Kampala, Uganda

**Keywords:** Human African Trypanosomiasis, Geographic information systems

## Abstract

**Introduction:**

Tsetse flies (*Glossina*) transmit *Trypanosoma brucei gambiense*, which causes gambiense human African trypanosomiasis (gHAT). As part of national efforts to eliminate gHAT as a public health problem, Uganda implemented a large-scale programme of deploying Tiny Targets, which comprise panels of insecticide-treated material which attract and kill tsetse. At its peak, the programme was the largest tsetse control operation in Africa. Here, we quantify the impact of Tiny Targets and environmental changes on the spatial and temporal patterns of tsetse abundance across North-Western Uganda.

**Methods:**

We leverage a 100-month longitudinal dataset detailing *Glossina fuscipes fuscipes* catches from monitoring traps between October 2010 and December 2019 within seven districts in North-Western Uganda. We fitted a boosted regression tree (BRT) model assessing environmental suitability, which was used alongside Tiny Target data to fit a spatiotemporal geostatistical model predicting tsetse abundance across our study area (~16 000 km^2^). We used the spatiotemporal model to quantify the impact of Tiny Targets and environmental changes on the distribution of tsetse, alongside metrics of uncertainty.

**Results:**

Environmental suitability across the study area remained relatively constant over time, with suitability being driven largely by elevation and distance to rivers. By performing a counterfactual analysis using the fitted spatiotemporal geostatistical model, we show that deployment of Tiny Targets across an area of 4000 km^2^ reduced the overall abundance of tsetse to low levels (median daily catch=1.1 tsetse/trap, IQR=0.85–1.28). No spatial–temporal locations had high (>10 tsetse/trap/day) numbers of tsetse compared with 18% of locations for the counterfactual.

**Conclusions:**

In Uganda, Tiny Targets reduced the abundance of *G. f. fuscipes* and maintained tsetse populations at low levels. Our model represents the first spatiotemporal geostatistical model investigating the effects of a national tsetse control programme. The outputs provide important data for informing next steps for vector control and surveillance.

**WHAT IS ALREADY KNOWN ON THIS TOPIC**

- Small panels of insecticide-treated fabric, called Tiny Targets, are used to attract and kill riverine tsetse, the vectors of *Trypanosoma brucei gambiense,* which causes gambiense human African trypanosomiasis (gHAT). In large-scale (250–2000 km^2^) trials conducted in five countries, deployment of Tiny Targets reduced the densities of tsetse by between 60% and >90%.

**WHAT THIS STUDY ADDS**

- We report an analysis of, and data from, a large-scale (~4000 km^2^) national tsetse control programme, implemented in Uganda to eliminate gHAT as a public health problem. We found that Tiny Targets reduced tsetse abundance across the study period (2011–2019) and maintained densities at low (<1 tsetse/trap/day) levels. We produce maps that detail spatial variations in tsetse abundance in response to vector control.

**HOW THIS STUDY MIGHT AFFECT RESEARCH, PRACTICE OR POLICY**

- In 2022, Uganda received validation from the WHO that it had eliminated gHAT as a public health problem. The large-scale deployment of Tiny Targets contributed to this achievement. Our findings suggest that Tiny Targets are an important intervention for eliminating gHAT in other countries.

## Background

 Human African trypanosomiasis (HAT), commonly called sleeping sickness, is caused by subspecies of *Trypanosoma brucei* transmitted by tsetse flies (*Glossina*). In West and Central Africa, gambiense HAT (gHAT) is caused by *T. b. gambiense* transmitted by riverine species of tsetse (eg, *G. palpalis palpalis*, *G. fuscipes*). In East and Southern Africa, *T. b. rhodesiense* transmitted by savanna species of tsetse (eg, *G. morsitans morsitans*, *G. pallidipes*) causes rhodesiense HAT (rHAT). Both diseases are fatal without medical intervention. Uganda is the only country where both forms of HAT occur.^1^

The last major epidemic of HAT in Uganda occurred in the last~20 years of the 20th century when political and economic upheavals disrupted national control programmes. Between 1990 and 1999, Uganda reported an average of 1384 cases/year (range: 971–2066) of gHAT and 516 cases/year (178–1417) of rHAT.^2^ Since then, numbers of both forms of HAT have declined. In the last 5 years for which data are available (2018–2022), there have been a total of four cases of gHAT and 13 cases of rHAT reported in Uganda. The dramatic decline in gHAT has been achieved through mass screening and treatment of human cases supported by the deployment of Tiny Targets to control tsetse.^3^ For rHAT, the decline has been achieved through mass treatment of cattle with trypanocides and insecticides because Ugandan cattle are important reservoir hosts for *T. b. rhodesiense*^4^ and cattle form the main source of a tsetse’s diet.

The achievements of Uganda over the past 20 years are part of a larger continental effort, led by the WHO, to eliminate gHAT as a public health problem by 2020 and eliminate transmission by 2030. Uganda’s achievement of the first goal was ratified by the WHO in May 2022.^5^ The second goal is defined as the ‘reduction to zero of the incidence of infection in a defined geographical area, with minimal risk of reintroduction, as a result of deliberate efforts’; this target will involve 15 endemic countries by 2030.^6^

For the last decade, deployment of Tiny Targets has formed an important part of Uganda’s strategy to control gHAT. Tiny Targets are small panels composed of blue cloth (25×25 cm) flanked by a panel (25×25 cm) of black netting. The cloth and netting are impregnated with insecticide; tsetse are attracted visually to the target and die on contacting.^7^ In Uganda, Tiny Targets are deployed at a density of 20 targets per linear kilometre along the rivers and streams where riverine tsetse concentrate. This intervention reduces the density of tsetse populations by 60%–99%.^7^,^11^ Epidemiological models^12 13^ and empirical evidence^10 11 14^ suggest that this reduction is sufficient to interrupt transmission. The very first trials of Tiny Targets were carried out in Uganda in 2011^7^ and from an initial trial covering ~250 km^2^ the intervention grew to an operation of ~4000 km^2^ across seven districts. At its peak, Uganda was implementing the largest national tsetse control operation in Africa. Tiny Targets are also making important contributions to the elimination of gHAT in Côte d’Ivoire,^9^ Chad,^10^ Democratic Republic of Congo (DRC)^15^ and Guinea.^11^

The large-scale deployment of Tiny Targets in Uganda has been accompanied by an extensive monitoring programme comprising a network of entomological sentinel sites, where pyramidal tsetse traps are used to quantify the abundance of tsetse before and after targets were deployed.^7 8^ This monitoring programme has produced a decade of data on the distribution and abundance of tsetse in and near the places where Tiny Targets have been deployed in North-West Uganda.

Prior analyses of the impact of targets in Uganda^7 8^ and elsewhere^9^,^1115^ have shown that the reductions in density varied between 55% and >99%. The causes of this variation are unknown, but we hypothesise that the differences are due, in part, to underlying environmental factors. Previous estimates compared catches from individual sites before and after an intervention and could not consider what happened in places where we did not sample. The spatial and temporal scale of the monitoring data accompanying control operations in Uganda provides a unique opportunity to quantify the impact of a large-scale tsetse control programme and assess the relative contributions of Tiny Targets and environmental factors to the reduction in tsetse abundance throughout Uganda, in areas which were not measured empirically. To do this, we first developed temporally varying estimates of tsetse habitat suitability within North-Western Uganda, using preintervention entomological survey data, remotely sensed environmental data and a species distribution model (SDM). Suitability outputs were then combined with data from subsequent postintervention surveys to quantify the impact of both environmental change and vector control on the abundance of *Glossina fuscipes fuscipes*, the main g-HAT vector in Uganda, through a spatiotemporal geostatistical modelling approach.

## Methods

### Patient and public involvement

No patients were involved in this study.

### Study area

**Figure 1:**
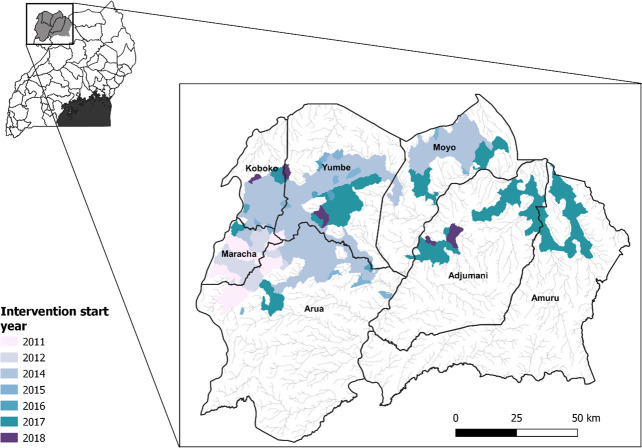
Districts within North-Western Uganda in which tsetse monitoring was performed, alongside watersheds controlled by Tiny Targets. The seven districts which form the basis of this analysis cover a total of ~16 419 km^2^, combined coverage of intervention areas is ~4000 km^2^. Colours represent the years in which the Tiny Target intervention was first rolled out within each district, constructed using data from Bessell *et al*.^14^ Targets were replaced every 6 months. Map produced using QGIS V.3.16.5.^62^

Trapping was performed to quantify the impact of Tiny Targets on the abundance of tsetse.^7 16^ Between October 2010 and December 2019, pyramidal traps^17^ were deployed within seven districts in North-Western Uganda to monitor the abundance of *G. f. fuscipes*.^7 18^ Traps were deployed for 1–4 consecutive days (median 2 days), with tsetse collected and counted at 24-hour intervals.^7 19^ Monitoring and control activities were scaled-up in phases according to need and available funding. Therefore, initial deployment of traps and targets varied between and within districts. The year in which intervention was initiated in each district is displayed in figure 1, using watersheds as a nominal metric of coverage^14^—further detail regarding survey dates and distribution are provided within online supplemental methods.

### Identification of intervention areas

Tiny Targets are deployed along riverbanks within the intervention area two times per year. They are deployed at 100 m intervals along each bank, that is, 20 targets per kilometre of river, with their location recorded using global positioning systems (GPSs). We generated a 30×30 m resolution grid for the entire study area and the distance from the centre of each grid cell to the nearest Tiny Target was calculated per deployment period. We assumed that targets were effective for 6 months following their deployment (online supplemental table 1) based on other work.^7^ These distance surfaces were used to produce a categorical variable classifying each gridded pixel as (1) within 500 m of a target, (2) >500 m but <5000 m or (3) >5000 m from a target.^15^ Henceforth, the three categories are termed inside, edge and outside, respectively.

### Tsetse data for model

Geographic locations of monitoring traps were recorded using GPSs alongside additional variables outlined in online supplemental table 2. From collected records, we produced two separate datasets: one for use in an SDM predicting habitat suitability and another for use in a geostatistical spatiotemporal modelling framework predicting tsetse abundance over time. The main differences between the data requirements for the two models were that the spatiotemporal geostatistical model was fitted to count data of tsetse from all traps, and the SDM used presence–absence data with observations being limited to traps considered to be unaffected by the intervention, that is, operated before any intervention or ≥5 km from a Tiny Target. As the geographical extent of the intervention increased, some traps classed initially as being ‘non-intervention’ transitioned to an ‘intervention’ status.

### Assembling explanatory variables

Gridded surfaces for temperature, elevation and vegetation were assembled for the seven districts (extent shown in figure 1, covariates summarised in table 1). These variables have been shown previously to influence the distribution of tsetse.^20^,^24^ Covariates were generated from remotely sensed satellite imagery collected at a spatial resolution of 30×30 m during the dry season—December–February. Cloud coverage and clear scene availability affected our capability to collate imagery for other times of year. Where available, temporally varying covariates were collated annually. Non-temporally varying covariates (elevation, distance to rivers, slope) were included as synoptic surfaces (table 1). To account for tsetse dispersal,^25^ a buffer with a radius of 150 m was used to produce a smoothed mean covariate derived from averaging all 30 m cells across 300 m of the true sample location. An overview of the covariate production process is provided in the online supplemental methods.

**Table 1:** Description and source of covariates used within the presence–absence modelling framework

Temporal resolution	Covariate (unit)	Rationale (reference)	Source
Synoptic	Elevation (metres)	22 23	Shuttle Radar Topography Mission (SRTM)^63^
	Distance to rivers (metres)	25	Derived from SRTM elevation data^63^
	Slope (percentage gain)		Derived from SRTM elevation data^63^
Annual (2011–2019) (dry season)	Land surface temperature day (mean) (°C)	20 21	Derived from Landsat 5^64^ Derived from Landsat 8^65^
	Normalised Difference Vegetation Index (−1 to 1)	24	Derived from Landsat 5^64^ Derived from Landsat 8^65^
	Proportion of vegetation (0–1)	24	Derived from Landsat 5^64^ Derived from Landsat 8^65^

### Species distribution model (SDM)

To estimate tsetse densities in locations where no sampling was performed, we produced annual estimates of habitat suitability using a presence–absence SDM. SDMs predict the distribution of a species across a landscape^26^ by combining information on species occurrence with environmental variables (covariates) at the same location.^27^ Using the species occurrence dataset and covariates detailed above, we constructed a presence–absence BRT model using the ‘caret’ package within R (V.3.5.1).^28 29^

BRTs are a machine learning algorithm, which combine both regression trees and boosting (iteratively combining a group of simple models) to build a linear combination of many trees^30^ and have been used to predict the distributions of a number of diseases and disease vectors.^1531^,^33^ The BRT method models a suitability index from 0 to 1 for tsetse based on the values of environmental covariates at the locations corresponding to presence–absence inputs.^26^ In this instance, ‘presence’ and ‘absence’ records refer to sampling locations where tsetse were caught or not. The absence of tsetse from a monitoring trap may reflect the true absence of tsetse or that tsetse were present but the trap failed to catch any. Accordingly, ‘absence’ records are commonly referred to as ‘background points’ and serve the purpose of exposing the model to locations where the species is presumed to be absent.^34^ Further information regarding the covariates used within the model, model fitting and methods of model evaluation can be found in online supplemental methods.

### Spatiotemporal model

To evaluate the impact of Tiny Targets and environmental variables on tsetse, a geostatistical spatiotemporal model was constructed using catch data. Data for this model included repeat catches from sites within the same year. Prior to constructing the spatiotemporal model, a series of exploratory plots and analyses were performed. An empirical variogram was constructed to test for spatial autocorrelation and to obtain starting parameter values for use within the model; this variogram was fitted using the ‘PrevMap’ R package.^35^ A Pearson’s χ^2^ test was performed to determine the level of dispersion within the data using the ‘msme’ R package.^36^ Given the high number of zero catches, our a priori assumption was a high level of overdispersion. This was confirmed (dispersion=4.79), and therefore a negative binomial distribution was most appropriate for modelling.^37^ As trapping success is highly variable, and zeros may arise due to either the true absence of the species, or due to trapping failure, we opted to model excess zeros independently through use of a zero-inflated negative binomial (ZINB) model and a type 1 likelihood. A type 1 likelihood accounts for two different types of zeros within the dataset: *structural* or true zeros, which represent the true absence of tsetse in a location, and *sampling* zeros, where a zero is recorded as a reflection of chance.^38^ Further information regarding model construction, including a full model description, can be found in online supplemental methods.

### Model fitting and validation

Models were fit through integrated nested Laplace approximations (INLA) and a stochastic partial differential equation representation of the Gaussian-Markov random field approximation to the Gaussian process model, based on a Matérn covariance function, using the R INLA package.^39^ To evaluate the significance of fixed and random effects on tsetse abundance, an iterative process was performed where varying combinations of fixed and random effects were used within models to identify the optimal model construction.

A priori, we hypothesised that the effect ‘suitability’, that is, the output of the BRT environmental suitability model, would be positively associated with the catch of tsetse. We hypothesised further that the ‘intervention’ effect, that is, proximity to Tiny Targets, would have a negative association on tsetse abundance. An interaction term between ‘suitability’ and ‘intervention’ was also included in the fitting process. Temporal effects were included in the form of ‘season’, a categorical ‘wet’ and ‘dry’ variable, to investigate temporal changes in abundance and through the inclusion of an autoregressive process of order 1, that is, AR(1). It was hypothesised that there will be seasonal changes in abundance, with a higher abundance of tsetse being observed in the wet season.^7 40^

Measures of the goodness of fit for the spatiotemporal model were obtained using the deviance information criterion (DIC). The DIC is a Bayesian generalisation of the Akaike information criterion, where models are penalised by their deviance and the number of parameters included.^41^ Using the variables identified from the model with the lowest DIC, we fitted a separable geostatistical spatiotemporal model described fully in online supplemental methods. The INLA approach does not allow for the combined fitting of a regression model for the zero-inflation probability of the zero-inflated model, therefore an additional function (‘pred.zinb’) was defined to apply the zero-inflation probability to a posterior sample (1000 draws) of non-zero-inflated data derived from the negative binomial model.^42^ Model validation was performed using a spatial leave-one-out cross-validation (SLOO-CV) approach, based on an adaptation of methods described in Le Rest *et al* and Lucas *et al*,^43 44^ and described further within online supplemental methods. Validation statistics included assessing the correlation between the predicted and observed tsetse densities through summaries of the root mean square error (RMSE) and the mean absolute error (MAE).^45^

Posterior predictive distributions (1000 draws) were simulated for each 30×30 m cell to determine the probability of tsetse catches exceeding predefined abundance categories defined as *low*, 0–1 flies; *medium*, >1–10 flies; and *high*, >10 flies. These categories were determined by discussion with field entomologists regarding policy-relevant values. The number of draws within each category was used to produce probabilities for each cell, low (pL), medium (pM), and high (pH) respectively, and each category was assigned a predictive score using the log-odds. Following Lowe *et al*,^46^ we used the receiver operating characteristic (ROC) to define optimal probability thresholds for assigning a final category to each cell by comparing the predictive score with the observed class, using the ‘ROCit’ R package.^47^ The probabilistic results were mapped using a ternary plotting technique^48^ and the ‘tricolore’ R package^49^ to visualise category certainty. Within the maps, the predicted category for each cell was expressed as a colour determined by a combination of the three probabilities assigned, with colour saturation used to indicate the associated certainty. Maps of the final category per cell were produced using QGIS and threshold values obtained from the ROC curves.

### Counterfactual analysis

To estimate the relative contribution of Tiny Targets to changes in tsetse abundance, a counterfactual analysis was performed using a 50% random sample of the longitudinal trapping data, that is, 4180 trap-month records. Using the optimal spatiotemporal model, all covariate values were held fixed except for the categorical intervention variable. The predicted mean flies per trap day was then compared for two predictions, where the value assigned to the intervention variable was changed:

1. 50% of records were assigned the intervention category ‘inside’ for the purpose of prediction and estimates of the mean daily catch of tsetse were generated for these locations.
2. In a different model run, the same 50% of records were assigned the intervention category ‘outside’, and estimates of the mean daily catch were generated for these locations.

To compare the abundances predicted by the original and counterfactual models, we quantified changes in the frequency of catches in the low, medium and high catch categories.

## Results

### Tsetse occurrence data

**Figure 2:**
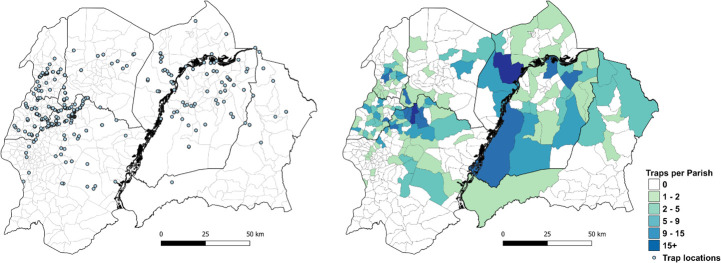
Location (left) and number of tsetse traps per parish (right), North-Western Uganda. Parish administrative boundaries obtained from www.GADM.org. Map produced using QGIS V.3.16.5.^62^

The dataset comprised 31 426 records from 569 locations sampled between October 2010 and December 2019 (figure 2) (online supplemental file 2). A total of 52 544 tsetse were captured over 31 553 trapping days (mean 1.67 flies/trap/day across all locations) (online supplemental table 3).

After spatial and temporal aggregation to retain one record per 30×30 m cell per year (presence at one time point replaces absence at another time within the same year), 538 unique location-year records situated outside the intervention area remained: 376 presence and 162 absence. The number of records per year is provided in online supplemental table 4. We sampled predominantly where we presumed tsetse to be present and so locations outside the intervention area reporting absences are relatively few.

### Habitat suitability maps

A BRT model was fitted to presence–absence data from 2010 to 2019 obtained outside the intervention area. Optimal values of the BRT model parameters, based on minimising the Brier score, were number of trees=350, interaction depth=29 and shrinkage=0.1. The evaluation measures for the resulting optimal BRT are as follows: AUC=0.81 and Brier score=0.21, representing a moderate model fit. The specificity and sensitivity of the model were 0.86 and 0.59, respectively, indicating a greater ability to identify absence records correctly (high specificity) and a greater error when predicting presence locations (low sensitivity). The BRT model may be calibrated to favour optimising sensitivity or specificity or calibrated to equally prioritise both. For this study, we used default settings for balancing sensitivity and specificity.

**Figure 3:**
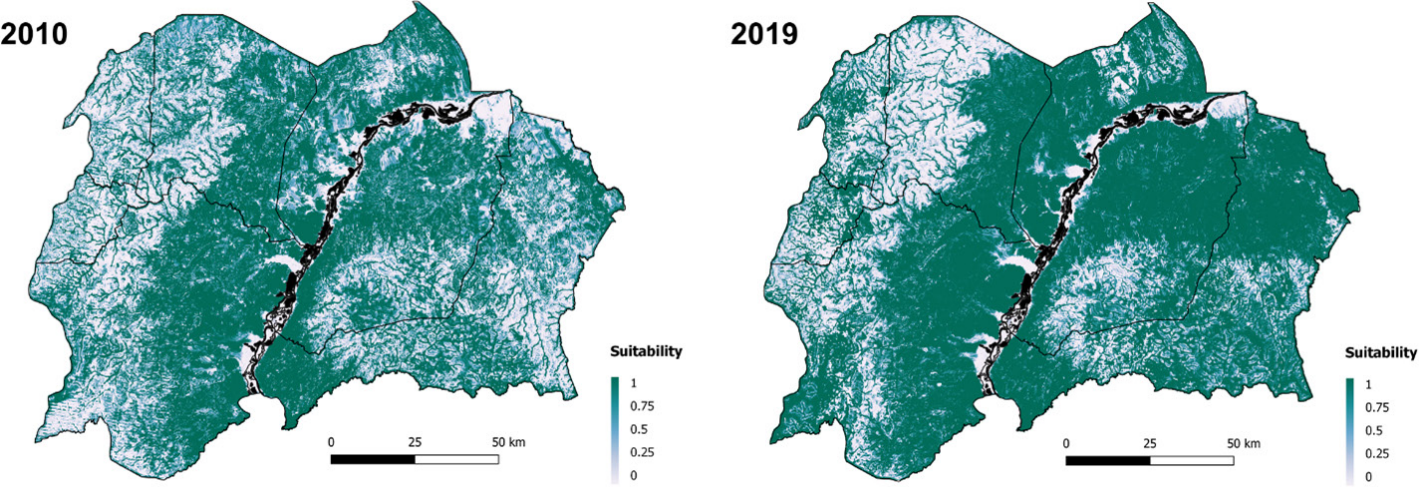
Predicted habitat suitability for *Glossina fuscipes fuscipes* within North-Western Uganda, for 2010 and 2019. Dark green locations indicate areas of higher environmental suitability; whiter areas indicate areas of lower environmental suitability. Map created using QGIS V.3.16.5.^62^

The relative importance of each of the environmental variables included, with respect to their contribution to the final BRT, is presented in online supplemental table 5. Elevation (m), Normalised Difference Vegetation Index (NDVI) and distance to rivers (m) were equally important contributors (19.14%, 18.57% and 18.47%, respectively), to variation in suitability. Using the fitted BRT model, predictions of habitat suitability for tsetse were made for each 30×30 m cell for 8 years: 2010, 2013–2019. Maps showing suitability for the years 2010 and 2019 are presented in figure 3; maps for other years are provided in online supplemental figure 1. Areas of high suitability follow rivers, vegetated and high-elevation areas neighbouring the Albert Nile and parts of Amuru and Adjumani districts. Spatial trends are visible across years. For parts of Amuru and Adjumani district, predicted suitability was greater in 2019 than 2010; however, this may be an artefact of the low sensitivity of the model (0.59).

### Spatiotemporal model

After collating all sampling records, a 100-month continuous longitudinal dataset consisting of 416 sites was produced for North-Western Uganda. The series consisted of records from September 2011 to December 2019 and included 8360 trap-month combinations, that is, one trap-month combination is the total number of flies (count) reported at a trap, for a specific month. These data formed the basis of the spatiotemporal model.

To identify which fixed and random effects optimise the performance of the spatiotemporal model, a range of ZINB generalised linear geostatistical models were fitted to the series data, varying the fixed and/or random effects across models. A list of considered models, alongside their corresponding evaluation metrics (DIC, Watanabe-Akaike information criterion [WAIC] and CPO) is provided in online supplemental table 6. The optimal ZINB had an DIC of 35 538, compared with a median and maximum DIC of 36 100 and 37 064, respectively (online supplemental table 6).

The equation for the final model is as follows:


(1) $$\log\left(\eta \left(s,\;t\right)\right)=\begin{array}{l} \beta_{0}+\;\beta_{1}suitability_{s,t}+\;\beta_{2}intervention_{s,t}+\\ \beta_{3}season_{s,t}+\;\beta_{4}LTT_{s,t}+\;\beta_{5}suitability_{s,t}\times \\ intervention_{s,t}+\beta_{6}season_{s,t}\times intervention_{s,t}+\\ \beta_{7}LTT_{s,t}\times intervention_{s,t}+U_{s}+V_{s,t} \end{array}$$

where $\beta_{n},n=1,\ldots ,7$, represents the coefficients for each covariate associated with observations at location s at time t. The abbreviation *LTT* refers to a linear temporal trend, representing the sequential month in the continuous time series. *U*_*s*_ represents the spatially uncorrelated random effect (*site*_*ID*_), and *V*_*s,t*_ represents the spatially and temporally structured random effects fully defined in online supplemental methods.

Table 2 displays the posterior mean estimates and 95% Bayesian credible intervals (CrI) for the effects included within the optimal spatiotemporal model, fit to all observed locations and time periods (100-month series). Posterior distributions and CrI are visualised in online supplemental figure 2. Starting parameter and hyperparameter values for priors used within the model are provided within online supplemental table 7.

**Table 2:** Posterior mean estimates and credible intervals (CrI), alongside rate ratio estimates for the best fitting model (model 10, online supplemental table 6)

Variable	Mean	2.5% CrI	50% CrI	97.5% CrI	Rate ratio (95% CrI)
Suitability	0.089	−0.079	0.089	0.257	1.09 (0.92, 1.29)
Inside intervention area (<500 m)	0.420	−30.647	0.420	31.461	1.52 (4.90e^−14^, 4.61e^13^)
Edge of intervention area (>500 m, ≤5000 m)	0.769	−30.299	0.768	31.810	2.16 (6.94e^−14^, 6.53e^13^)
Outside of intervention area (>5000 m)	−0.036	−31.104	−0.036	31.006	0.96 (3.30e^−14^, 2.85e^13^)
Season: dry	–	–	–	–	–
Season: wet	0.011	−0.070	0.011	0.092	1.01 (0.93, 1.10)
LTT	−0.025	−0.027	−0.025	−0.023	0.98 (0.97, 0.98)
Suitability×inside of intervention area	–	–	–	–	–
Suitability×edge of intervention area	0.387	0.035	0.388	0.734	1.61 (0.96, 2.69)
Suitability×outside of intervention area	1.463	1.056	1.464	1.867	4.72 (2.66, 8.36)
Wet season×inside of intervention area	–	–	–	–	–
Wet season×edge of intervention area	0.044	−0.135	0.044	0.223	1.06 (0.81, 1.37)
Wet season×outside of intervention area	0.269	0.098	0.269	0.439	1.32 (1.03, 1.70)
LTT×inside of intervention area	–	–	–	–	–
LTT×edge of intervention area	−0.009	−0.012	−0.009	−0.005	0.97 (0.96, 0.97)
LTT×outside of intervention area	−0.009	−0.013	−0.009	−0.005	0.97 (0.96, 0.97)
Spatial range of the RF: ρ	0.097	0.095	0.097	0.715	–
Marginal SD of the RF: σ^2^	1.597	1.578	1.597	1.616	–
Size for negative binomial zero-inflated observations: α	0.704	0.699	0.704	0.710	–
Zero probability parameter for zero-inflated negative binomial: π	0.039	0.039	0.039	0.040	–

Blank (dashed) rows represent the reference category used with each interaction term.

LTT, linear temporal trend; RF, Gaussian random field.

We performed a SLOO-CV using the optimal spatiotemporal model configuration. In total, 415 separate submodels were produced (k–1). Each submodel was fitted to data excluding one randomly selected trap, and all traps within a radius of 0.097 decimal degrees from that trap. This radius was equivalent to ~12.8 km, the value defined by the posterior range of spatial autocorrelation (ρ) identified within the optimal model. The zero-inflation probability (π = 0.039, 95% CrI = 0.039, 0.040) was applied to a posterior sample consisting of 1000 draws of non-zero-inflated data within each model, and the mean predicted values across all 1000 draws for the excluded data were compared with the observed values. Resulting validation statistics include an RMSE of 8.021 and MAE of 6.048, lower than another published model from the region (RMSE=15.2).^16^

Through the conversion of posterior mean values into rate ratios, we determined the mean effect of each variable on the predictions. Habitat suitability is significantly and positively associated with fly catches outside of the intervention area (rate ratio (RR)=4.72, 95% CrI=2.66, 8.36). This effect weakens at the edge of intervention areas (RR=1.61, 95% CrI=0.96, 2.69) and inside the intervention areas (RR=1.09, 95% CrI=0.92, 1.29) (table 2). Other significant predictors include a linear temporal trend (significant negative effect, RR=0.98, 95% CrI=0.97, 0.98) and interactions between (1) season (wet) and intervention (significant positive effect, edge RR=1.06, 95% CrI=0.81, 1.37; outside RR=1.32, 95% CrI=1.03, 1.70), implying higher abundance of tsetse within the wet season and on the edge and outside of intervention areas compared with the dry season reference class and (2) intervention and the linear temporal trend (significant negative effect, RR=0.97, 95% CrI=0.96, 0.97) (table 2 and online supplemental figure 2).

**Figure 4:**
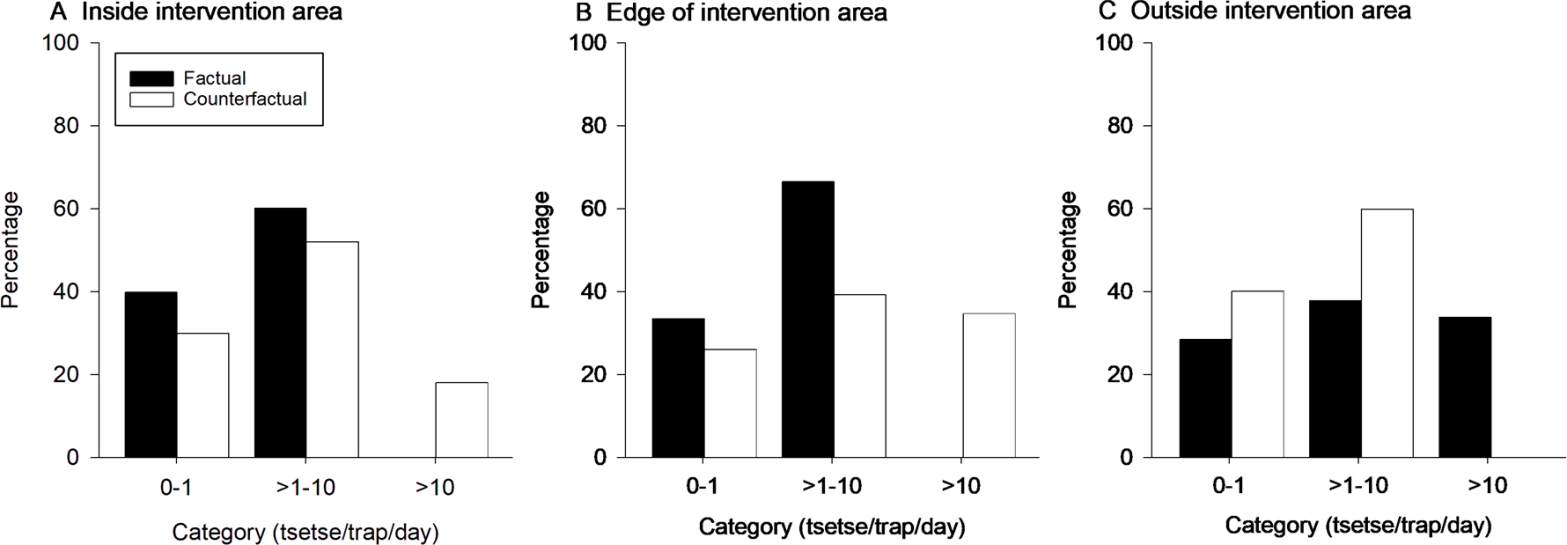
Frequency of catches (tsetse/trap/day) in low (0–1), medium (>1–10) and high (>10) categories for the factual and counterfactual models of tsetse abundance. (A) The percentage of traps containing low, medium and high number of flies for areas considered ‘inside’ (within 500 m of a target) the intervention area. (B) The percentage of traps for areas considered on the edge of the intervention area (>500 m but <5000 m from a target). (C) The percentage of traps for areas outside the intervention area (>5000 m from a target).

For traps inside the intervention areas, the counterfactual increased the median daily catch from 1.1 (IQR=0.93–1.28) to 2.3 (IQR=1.79–7.14) tsetse/trap. Conversely, for traps outside the intervention area, the counterfactual reduced the median daily catch from 3.8 (IQR=0.77–14.17) to 1.0 (IQR=0.94–1.28) tsetse/trap. Treating traps on the edge of the intervention area as being in areas affected by Tint Targets, the counterfactual increased the median daily catch increased from 1.2 (0.94–1.45) to 4.0 (0.95–19.66) tsetse/trap. The counterfactuals had marked effects on the frequency distribution of catches in the high categories (figure 4). For traps inside the intervention area, none were predicted to have mean daily catches of >10 tsetse/trap whereas for the counterfactual, 18.0% were in this category. Similarly, traps on the edge of the intervention area were predicted to have 34.7% in the high category if targets had not been deployed compared with none in their presence. Conversely, for traps outside the intervention area, 33.8% were predicted to have high catches compared with none for the counterfactual.

To determine threshold values for converting predictions into categorical estimates, a sample of 1000 draws were taken from the posterior distribution of the fitted model for each gridded cell. The probability of the prediction belonging to low, medium and high fly categories was produced (pL, pM and pH, respectively). These probabilities were compared with the true category from observed data, and ROC curves were generated (online supplemental figure 3). The model was able to distinguish between ‘low’ and ‘high’ categories with high accuracy, with AUC values of 0.83 and 0.91. In the operational setting, identifying these extremes will help prioritise area for control and/or monitoring. The ability to correctly identify the ‘medium’ category (between 1 and 10 flies) was lower than the other two, with an AUC value of 0.7 (online supplemental figure 3). Using the ROC curves, threshold values were obtained for assigning predictions to a specific category. If pL≥0.44, a cell was assigned the category ‘low’ abundance, if pL<0.44 and pM≥0.468, a cell was assigned the category ‘medium’ abundance, if pL<0.44 and pM*<*0.468, a cell was assigned the category ‘high’ abundance.

**Figure 5:**
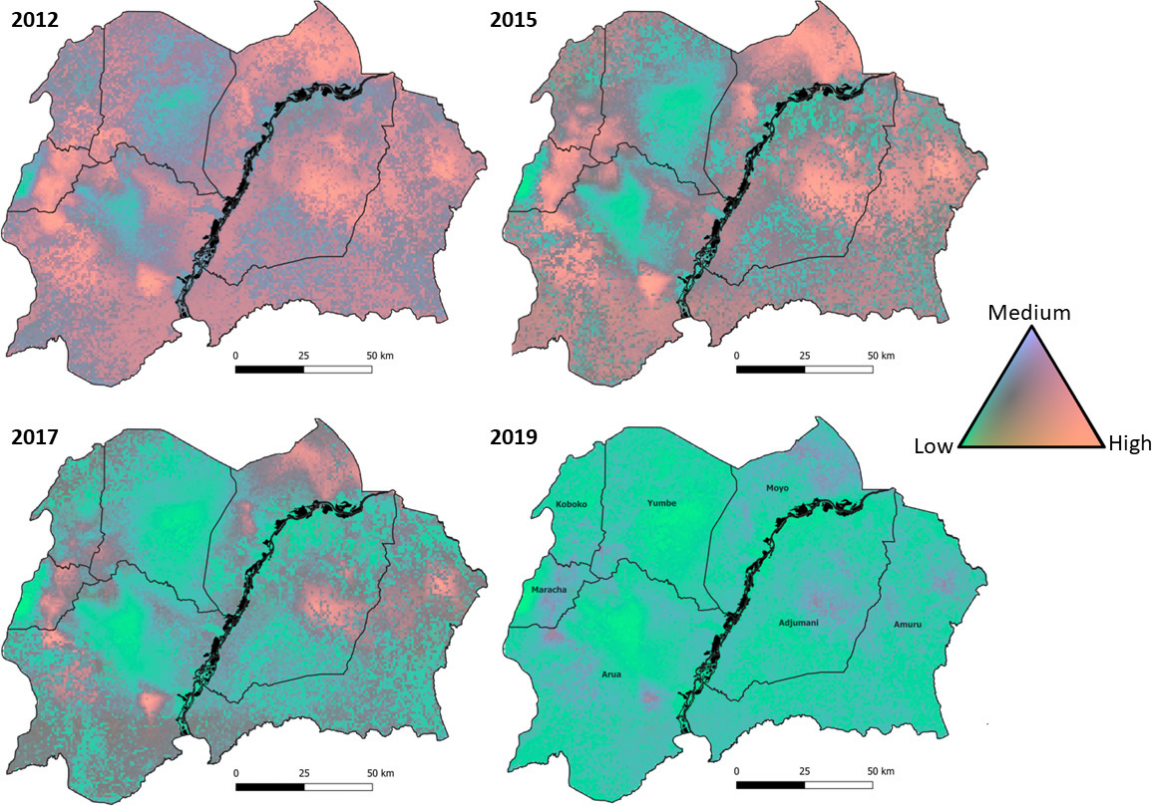
Comparison of categorised (low, medium and high) *Glossina fuscipes fuscipes* abundance during four time periods. The prediction period relates to February of each year (2012, 2015, 2017 and 2019). The continuous colour palette portrays the probabilities assigned to low-abundance, medium-abundance and high-abundance categories, with the low category representing 0 flies, medium 1–10 flies and high >10 flies. The greater the vibrancy, the more certain the prediction. Vibrant pink represents a high probability of a high abundance of tsetse, vibrant green represents a high probability of low abundance.

Predictions of tsetse abundance were produced for four periods: February 2012, 2015, 2017 and 2019, representing in turn (1) first trials of Tiny Targets,^7^ (2) first expansion from two to five districts,^8^ (3) second expansion to cover seven districts and (4) time of maximum coverage (2019). The outputs are visualised as ternary maps, displaying the assigned abundance category (low, medium and high) and associated certainty per gridded cell (figure 5). Plots showing the relative abundance of tsetse for each period are provided as online supplemental figure 4.

Comparing the categorical predictions from 2012 with those for 2019 (figure 5) highlights a striking reduction in tsetse abundance over time. Generally, many ‘high’ abundance areas transition to areas of ‘low’ abundance between the two periods, starting with Yumbe district (2015) and expanding to areas of Adjumani, Arua and Amuru in 2017. The overall relative distribution and abundance of tsetse, however, does not appear to change across years (online supplemental figure 4), despite the overall reductions in absolute abundance (online supplemental table 8). Persistent tsetse populations, although with a lower abundance, can be seen in North-West and Eastern Arua, Maracha, Central Adjumani and North-Eastern Amuru (figure 5 and online supplemental figure 4). Several of the relatively high abundance areas, such as Central Adjumani and Eastern Arua, are in places outside the 2019 intervention area (online supplemental figure 4). Maps representing the categorical prediction after applying the threshold values determined by the ROC curves are given as online supplemental figure 5; these maps can be used to inform additional monitoring and tsetse control operations.

## Discussion

We used a 100-month series of catches of tsetse from traps deployed at 416 sites across North-Western Uganda to produce species distribution and spatiotemporal models of the abundance of *G. f. fuscipes*, an important vector of sleeping sickness in Uganda and neighbouring countries (South Sudan, DRC). The SDM showed that the presence of tsetse was correlated negatively with elevation and positively with NDVI and proximity to rivers, in accord with previous studies.^15 16^ While temporal variation in habitat suitability occurs over the study period, few locations show trends of decreased suitability (areas in Maracha, Koboko, Arua and Yumbe when comparing 2013 and 2019 estimates) with some areas in Amuru and Adjumani showing increased suitability over time. In contrast, the spatiotemporal model showed that there was a decrease in the median and range of abundance of tsetse in areas where Tiny Targets were deployed. In particular, catches predicted to be high (>10 tsetse/trap/day) were absent in areas where Tiny Targets were deployed.

In 2019, as the national incidence of gHAT declined to record lows, Uganda commenced scale back of tsetse control operations in Maracha district, and currently (January 2024) there are no plans to deploy Tiny Targets in the future. This will mark the first time that no Tiny Targets are deployed in Uganda in over ten years. Our results describe the impact of a successful national tsetse control programme and also produce maps which identify places highly suitable for tsetse and where they may rebound fastest.

Our findings add to earlier smaller-scale studies showing that Tiny Targets are a highly cost-effective method of controlling gHAT vectors,^50 51^ leading to their adoption in national programmes to eliminate gHAT.^3^ We leverage one of the most data-rich longitudinal datasets of abundance of riverine tsetse in existence (31 553 trapping days), to produce a high-spatial resolution model of tsetse abundance. Our predictions span a ~16 000 km^2^ extent within which Tiny Targets were deployed over an area of ~4000 km^2^. This approach expanded on earlier work performed in select districts, and for one time period (2010).^16^ Here, we produce a separate model which considered both spatial and temporal variation, as well as the incorporation and assessment of intervention measures on abundance, through information on the deployment of Tiny Targets between 2011 and 2019. The spatiotemporal model outperforms that for the previous spatial analysis when looking at metrics of predictive power for known trapping locations, that is, RMSE of 8.02 flies versus 15.2. However, the RMSE for our model remains inflated due to the generation of mean estimates across posterior samples containing high numbers of zeros. Metrics looking at the accuracy of categorised predictions, that is, low (0–1 flies), medium (>1–10) and high (>10) indicate greater accuracy than direct counts (online supplemental figure 3).

By performing a counterfactual analysis using the fitted ZINB geostatistical model and varying the intervention category, we show that Tiny Targets reduced the catches of tsetse from monitoring traps between 2011 and 2019. Our results accord with analyses from trials of Tiny Targets in Uganda^7 8^ and suggest that implementation by District tsetse control teams was highly effective. Our results are also comparable to those from the DRC, where an 85.5% reduction was attributed to the deployment of Tiny Targets.^15^ In Chad, an even higher level of control (99.5%) was achieved,^10^ probably reflecting local agroecological differences. The most important of such differences was perhaps that the intervention in Chad was directed against a relatively small and isolated population of tsetse associated with a wetland, whereas in Uganda and DRC the tsetse population was distributed throughout a complex and extensive river network. The targets had a marked impact on the edge of the intervention area in accord with the high mobility of tsetse. Empirical evidence^8^ and theoretical models^52^ of the impact of Tiny Targets on tsetse populations predict impact in areas beyond where the targets are deployed.

Our analyses identified several ‘hotspots’ within the intervention areas where tsetse were predicted to be relatively abundant (online supplemental figure 4). Indeed, while the abundance of tsetse declined over time (figure 5), we did not see complete elimination of tsetse within areas which have been subject to prolonged control. Tiny Targets are deployed to reduce populations to a level where transmission is interrupted rather than eliminate tsetse themselves. Modelling analysis indicates a ~60% population suppression is required to achieve interruption of transmission within DRC.^53^

The predicted reduction of tsetse in areas where Tiny Targets were not deployed, for example, Central Adjumani and Southern Arua (figure 5), may be attributable to temporal changes not explicitly incorporated within our model but which were captured via the inclusion of a temporal random effect, that is, noise within the autoregressive order 1 model, and a linear temporal trend (RR=0.98, 95% CI=0.97–0.98). Additional temporally varying covariates to consider incorporating within future iterations of the model include human population density, climatic variables such as precipitation, and land-use change, which may further explain the temporal trends observed within the data.^54 55^ North-Western Uganda has experienced large levels of development within the last decade, primarily due to an influx of refugees resulting in land-use change, that is, degraded grasslands, woodlands and tree plantations^56 57^ and human population growth (averaging 3.4% between 2010 and 2020),^58^ among other factors. Despite these developments, our SDMs show that environmental factors have either remained the same or improved for tsetse.

Further research is required to determine the link between suitable tsetse habitat and/or tsetse abundance and the geographical distribution of reported gHAT cases. Prior work has shown that proximity to Tiny Targets reduces the risk of gHAT in North-Western Uganda.^16^ However, not all tsetse infested areas are areas of gHAT risk, with tsetse also transmitting trypanosome species pathogenic to livestock but not to humans.^19 59^ Modelling the spatiotemporal variation in gHAT risk requires not only the accurate quantification of the distribution and abundance of parasite, vector and host populations but also treatment seeking behaviour, that is, proportion seeking diagnosis and treatment, as well as an understanding of diagnostic accessibility. Quantifying each of these factors would aid planning and implementation of interventions to eliminate transmission of gHAT.

Countries, which have eliminated gHAT or are preparing elimination dossiers for submission to WHO, need to identify and monitor remaining tsetse populations.^60^ The methods and predictions described here may be combined with estimates of geographic accessibility to provide a rationale for the placement of cost-effective sentinel monitoring sites to monitor and confirm tsetse population suppression, as demonstrated by Longbottom *et al*.^61^ Additionally, the models produced identify locations for which we have the least certainty regarding abundance of tsetse. Estimates of confidence aid the identification of areas where further baseline data may improve our understanding—quantifying this improves a process which was previously driven solely by expert opinion and ease of sampling.

## Conclusions

We show that a large-scale national programme of tsetse control, covering ~4000 km^2^ across seven districts, in which district-level teams deployed Tiny Targets, greatly reduced the overall abundance of tsetse and contributed to the elimination of gHAT as a public health problem in Uganda.

Tiny Targets reduced the abundance of tsetse in all areas where they were deployed. In places where the habitat and environment are highly suitable for tsetse, populations remained at low numbers, with no locations witnessing extinction of local populations. Such sites should be monitored for any rebound of tsetse and transmission of gHAT. Maps produced by this study can help to optimise surveillance strategies.

There was no clear and consistent decline in the environmental suitability for tsetse, suggesting that natural and anthropogenic change have had little impact on tsetse in North-Western Uganda over the last decade.

## Supplementary material

10.1136/bmjgh-2024-015374 online supplemental file 1

10.1136/bmjgh-2024-015374 online supplemental file 2

## Data Availability

All data relevant to the study are included in the article or uploaded as online supplemental information.

## References

[1] Simarro PP, Cecchi G, Paone M (2010). The Atlas of human African trypanosomiasis: a contribution to global mapping of neglected tropical diseases. Int J Health Geogr.

[2] World Health Organization (2021). The global health observatory: human african trypanosomiasis (sleeping sickness). https://www.who.int/data/gho/data/themes/topics/human-african-trypanosomiasis.

[3] Ndung’u JM, Boulangé A, Picado A (2020). Trypa-NO! contributes to the elimination of gambiense human African trypanosomiasis by combining tsetse control with “screen, diagnose and treat” using innovative tools and strategies. PLoS Negl Trop Dis.

[4] Welburn SC, Coleman PG, Maudlin I (2006). Crisis, what crisis? Control of Rhodesian sleeping sickness. Trends Parasitol.

[5] World Health Organization (2022). Benin, uganda and rwanda eliminate human african trypanosomiasis as a public health problem. https://www.who.int/news/item/24-05-2022-benin--uganda-and-rwanda-eliminate-human-african-trypanosomiasis-as-a-public-health-problem.

[6] World Health Organization (2021). Ending the Neglect to Attain the Sustainable Development Goals: A Road Map for Neglected Tropical Diseases 2021–2030, Ntuli MM Ed.

[7] Tirados I, Esterhuizen J, Kovacic V (2015). Tsetse Control and Gambian Sleeping Sickness; Implications for Control Strategy. PLoS Negl Trop Dis.

[8] Hope A, Mugenyi A, Esterhuizen J (2022). Scaling up of tsetse control to eliminate Gambian sleeping sickness in northern Uganda. PLoS Negl Trop Dis.

[9] Kaba D, Djohan V, Berté D (2021). Use of vector control to protect people from sleeping sickness in the focus of Bonon (Côte d’Ivoire). PLoS Negl Trop Dis.

[10] Mahamat MH, Peka M, Rayaisse J-B (2017). Adding tsetse control to medical activities contributes to decreasing transmission of sleeping sickness in the Mandoul focus (Chad). PLoS Negl Trop Dis.

[11] Courtin F, Camara M, Rayaisse J-B (2015). Reducing Human-Tsetse Contact Significantly Enhances the Efficacy of Sleeping Sickness Active Screening Campaigns: A Promising Result in the Context of Elimination. PLoS Negl Trop Dis.

[12] Rock KS, Torr SJ, Lumbala C (2017). Predicting the Impact of Intervention Strategies for Sleeping Sickness in Two High-Endemicity Health Zones of the Democratic Republic of Congo. PLoS Negl Trop Dis.

[13] Rock KS, Torr SJ, Lumbala C (2015). Quantitative evaluation of the strategy to eliminate human African trypanosomiasis in the Democratic Republic of Congo. *Parasites Vectors*.

[14] Bessell PR, Esterhuizen J, Lehane MJ (2021). Estimating the impact of Tiny Targets in reducing the incidence of Gambian sleeping sickness in the North-west Uganda focus. *Parasites Vectors*.

[15] Tirados I, Hope A, Selby R (2020). Impact of tiny targets on Glossina fuscipes quanzensis, the primary vector of human African trypanosomiasis in the Democratic Republic of the Congo. PLoS Negl Trop Dis.

[16] Stanton MC, Esterhuizen J, Tirados I (2018). The development of high resolution maps of tsetse abundance to guide interventions against human African trypanosomiasis in northern Uganda. *Parasites Vectors*.

[17] Gouteux JP, Lancien J (1986). The pyramidal trap for collecting and controlling tsetse flies (Diptera: Glossinidae). Comparative trials and description of new collecting technics. Trop Med Parasitol.

[18] Lindh JM, Goswami P, Blackburn RS (2012). Optimizing the colour and fabric of targets for the control of the tsetse fly Glossina fuscipes fuscipes. PLoS Negl Trop Dis.

[19] Cunningham LJ, Lingley JK, Tirados I (2020). Evidence of the absence of human African trypanosomiasis in two northern districts of Uganda: Analyses of cattle, pigs and tsetse flies for the presence of Trypanosoma brucei gambiense. PLoS Negl Trop Dis.

[20] Are EB, Hargrove JW (2020). Extinction probabilities as a function of temperature for populations of tsetse (Glossina spp.). PLoS Negl Trop Dis.

[21] Phelps RJ, Burrows PM (1969). Puparial duration in Glossina morsitans orientalis under conditions of constant temperature. Entomol Exp Applic.

[22] Rogers DJ, Robinson TP, Maudlin I, Holmes PH, Miles MA (2004). The Trypanosomiases.

[23] Albert M, Wardrop NA, Atkinson PM (2015). Tsetse fly (G. f. fuscipes) distribution in the Lake Victoria basin of Uganda. PLoS Negl Trop Dis.

[24] Esterhuizen J, Njiru B, Vale GA (2011). Vegetation and the Importance of Insecticide-Treated Target Siting for Control of Glossina fuscipes fuscipes. PLoS Negl Trop Dis.

[25] Rogers D (1977). Study of a Natural Population of Glossina fuscipes fuscipes Newstead and a Model of Fly Movement. J Anim Ecol.

[26] Elith J, Leathwick JR (2009). Species Distribution Models: Ecological Explanation and Prediction Across Space and Time. Annu Rev Ecol Evol Syst.

[27] Barry S, Elith J (2006). Error and uncertainty in habitat models. J Appl Ecol.

[28] Kuhn M (2020). caret: Classification and Regression Training. R package version 6.0-86.

[29] R Core Team (2020). R version 3.5.1 (2018-07-02) -- “feather spray.”.

[30] Elith J, Leathwick JR, Hastie T (2008). A working guide to boosted regression trees. J Anim Ecol.

[31] Longbottom J, Browne AJ, Pigott DM (2017). Mapping the spatial distribution of the Japanese encephalitis vector, Culex tritaeniorhynchus Giles, 1901 (Diptera: Culicidae) within areas of Japanese encephalitis risk. *Parasites Vectors*.

[32] Wiebe A, Longbottom J, Gleave K (2017). Geographical distributions of African malaria vector sibling species and evidence for insecticide resistance. Malar J.

[33] Mylne AQN, Pigott DM, Longbottom J (2015). Mapping the zoonotic niche of Lassa fever in Africa. Trans R Soc Trop Med Hyg.

[34] Phillips SJ, Dudík M, Elith J (2009). Sample selection bias and presence-only distribution models: implications for background and pseudo-absence data. Ecol Appl.

[35] Giorgi E, Diggle P (2017). PrevMap: An R Package for Prevalence Mapping. J Stat Softw.

[36] Hilbe J, Robinson A (2018). Msme: functions and datasets for “methods of statistical model estimation” version 0.5.3. p. Functions and datasets from hilbe, J.M., and robinson, A.P. 2013. Methods of statistical model estimation.

[37] Lindén A, Mäntyniemi S (2011). Using the negative binomial distribution to model overdispersion in ecological count data. Ecology.

[38] Arab A (2015). Spatial and Spatio-Temporal Models for Modeling Epidemiological Data with Excess Zeros. Int J Environ Res Public Health.

[39] Lindgren F, Rue H (2015). Bayesian Spatial Modelling with R-INLA. J Stat Softw.

[40] Wamwiri FN, Changasi RE (2016). Tsetse Flies (Glossina) as Vectors of Human African Trypanosomiasis: A Review. Biomed Res Int.

[41] Spiegelhalter DJ, Best NG, Carlin BP (2002). Bayesian Measures of Model Complexity and Fit. J R Stat Soc Ser B.

[42] Liang D (2015). Predictions for zeroinflated models: R INLA discussion group. https://groups.google.com/g/r-inla-discussion-group/c/KywB6rpWEyQ.

[43] Le Rest K, Pinaud D, Monestiez P (2014). Spatial leave‐one‐out cross‐validation for variable selection in the presence of spatial autocorrelation. Glob Ecol Biogeogr.

[44] Lucas T, Python A, Redding D (2020). Graphical outputs and Spatial Cross-validation for the R-INLA package using INLAutils. arXiv.

[45] Chai T, Draxler RR (2014). Root mean square error (RMSE) or mean absolute error (MAE)? – Arguments against avoiding RMSE in the literature. Geosci Model Dev.

[46] Lowe R, Coelho CA, Barcellos C (2016). Evaluating probabilistic dengue risk forecasts from a prototype early warning system for Brazil. Elife.

[47] Khan MRA, Brandenburger T (2020). ROCit: performance assessment of binary classifier with visualization. p. R package version 2.1.1.

[48] Jupp TE, Lowe R, Coelho CAS (2012). On the visualization, verification and recalibration of ternary probabilistic forecasts. Phil Trans R Soc A.

[49] Schöley J, Kashnitsky I (2020). Tricolore: a flexible color scale for ternary compositions. Version: 1.2.2. p. A flexible color scale for ternary compositions with options for discretization, centering and scaling.

[50] Shaw APM, Tirados I, Mangwiro CTN (2015). Costs of using “tiny targets” to control Glossina fuscipes fuscipes, a vector of gambiense sleeping sickness in Arua District of Uganda. PLoS Negl Trop Dis.

[51] Rayaisse J-B, Courtin F, Mahamat MH (2020). Delivering “tiny targets” in a remote region of southern Chad: a cost analysis of tsetse control in the Mandoul sleeping sickness focus. Parasit Vectors.

[52] Vale GA, Hargrove JW, Hope A (2024). Modelled impact of Tiny Targets on the distribution and abundance of riverine tsetse. PLoS Negl Trop Dis.

[53] Rock KS, Pandey A, Ndeffo-Mbah ML (2017). Data-driven models to predict the elimination of sleeping sickness in former Equateur province of DRC. Epidemics.

[54] Mwanakasale V, Songolo P (2011). Disappearance of some human African trypanosomiasis transmission foci in Zambia in the absence of a tsetse fly and trypanosomiasis control program over a period of forty years. Trans R Soc Trop Med Hyg.

[55] Reid RS, Kruska RL, Deichmann U (2000). Human population growth and the extinction of the tsetse fly. Agric Ecosyst Environ.

[56] Bernard B, Aron M, Loy T (2022). The impact of refugee settlements on land use changes and vegetation degradation in West Nile Sub-region, Uganda. Geocarto Int.

[57] International Bank for Reconstruction and Development, The World Bank, and The Food and Agriculture Organization of the United Nations (2019). Rapid assessment of natural resource degradation in refugee impacted areas in Northern Uganda: technical report.

[58] The World Bank (2021). Population growth (annual %) - Uganda. https://data.worldbank.org/indicator/SP.POP.GROW?end=2020&locations=UG&start=2010.

[59] Opiro R, Opoke R, Angwech H (2021). Apparent density, trypanosome infection rates and host preference of tsetse flies in the sleeping sickness endemic focus of northwestern Uganda. BMC Vet Res.

[60] Solano P (2021). Need of entomological criteria to assess zero transmission of gambiense HAT. PLoS Negl Trop Dis.

[61] Longbottom J, Krause A, Torr SJ (2020). Quantifying geographic accessibility to improve efficiency of entomological monitoring. PLoS Negl Trop Dis.

[62] QGIS.org (2021). QGIS geographic information system. http://www.qgis.org/.

[63] U.S. Geological Survey (2000). USGS eros archive - digital elevation - shuttle radar topography mission (SRTM) 1 Arc-second global. https://www.usgs.gov/centers/eros/science/usgs-eros-archive-digital-elevation-shuttle-radar-topography-mission-srtm-1-arc?qt-science_center_objects=0#qt-science_center_objects.

[64] U.S. Geological Survey Landsat-5 imagery courtesy of the U.S. Geological survey.

[65] U.S. Geological Survey Landsat-8 imagery courtesy of the U.S. Geological survey.

